# Effect of DFIG control parameters on small signal stability in power systems

**DOI:** 10.1038/s41598-023-29278-5

**Published:** 2023-02-11

**Authors:** Liu Qi, Wu jiahui, Wang Haiyun, Zhang Hua, Yang Jian

**Affiliations:** 1 College of Electrical Engineering, Xinjiang University, Urumqi, 830047 CO People’s Republic of China; 2 CGN New Energy Investment (Shenzhen) Co., Ltd., Xinjiang Branch, Xinjiang, 830011 China

**Keywords:** Energy science and technology, Engineering

## Abstract

The doubly-fed induction generator (DFIG) with virtual inertia control and reactive damping control gives a renewable energy generation system inertia and damping characteristics similar to those of a thermal power plant, and the parameters of the control strategy have a direct impact on the small-signal stability of the system. This paper firstly introduces the operating characteristics and control strategies of DFIG-based damping control and virtual inertia control, establishes a small-signal model of the control-based DFIG integrated interconnected system, and investigates the effects of virtual inertia and reactive damping values on the small-signal stability of the system; then, the maximum damping ratio of the interval oscillation mode in small disturbance analysis is taken as the optimization objective, and the control parameters are the optimization variables. An optimization method of inertia and damping parameters is established for improving the small disturbance stability of the system. The results show that the optimization procedure could improve the damping ratio of the interval oscillation mode while ensuring the system frequency. The effects of virtual inertia and reactive damping values on the small signal stability of the system are investigated, and an optimal allocation model and method for virtual inertia used to improve the small disturbance stability of the system is proposed.

## Introduction

New energy generation is gradually replacing fossil energy generation in the grid. Power electronics connect most new energy generation to the grid and disconnect during operation. Renewable energy generation has minimal inertia. Thus, ordinary renewable energy producing equipment cannot regulate power supply frequency caused by grid oscillations^1^. Power system inertia decreases. This threatens power system frequency stability^2^.

Virtual inertia control is to use the kinetic energy stored in the rotor of the wind turbine to increase the electromagnetic power output of the inverter^3^ and slow down the frequency drop^4^ in a short time . In terms of ways to improve the control method, The author of^5^ proposes a general virtual inertia control strategy that can regulate the inertia power provided by the wind turbine by modifying the corresponding virtual inertia control coefficients. Some researchers have proposed an online under-frequency load shedding (UFLS) strategy considering virtual inertia control of wind turbines^6^. It can use the FM potential of wind farms to their fullest and optimize under-frequency load shedding by using fuzzy techniques to cut loads more accurately and speed up frequency recovery. The way that virtual inertia is spread out in the system can also affect how stable the system is. Some researchers have pointed out that the optimal placement of virtual inertia can be studied from two perspectives: frequency stability and small-signal stability. Some researchers have used the performance metric of the parametric number of parameters to show the stability of the system^7^. The optimal inertia layout problem is turned into a non-convex problem, and a way to find a locally best solution is shown. However, because the effect of random fluctuations in measurements during modeling is not controllable and the damping coefficient is not a decision variable in the problem setup^8^, the situation becomes complicated when considering extensions to more detailed system models and specifications. The paper^9^ proposes an optimization procedure to answer the question of how changes in inertia affect the stability of the power system and where it is most appropriate to place the device providing the virtual inertia. But the article doesn't take into account how the inertia system interacts with other control loops, and it's too complicated to solve the nonlinear problem. For practical purposes, a simpler approach is needed.

However, the wind turbine may affect the small disturbance stability of the system after the introduction of virtual inertia feedback link. The oscillation mode of a doubly-fed wind turbine after the introduction of virtual inertia has been analyzed. This has the potential to deteriorate the small disturbance stability of the system and to reduce the damping ratio of the inter-regional oscillation modes. In the case of insufficient damping, the inertia change of the system after disturbance may lead to severe transient frequency oscillations^10^. Many studies in recent years have shown that reactive power control with wavelet transform also plays an important role in improving the system stability. In^11^, the effect of active and reactive power control on the synchronous stability of the system was analyzed and it was found that reactive power damping control (RPDC) helps to counteract the side effects of virtual inertia control(VIC) on the damping of electromechanical oscillations. In^12^ author explored the role of RPDC through root trajectory analysis and simulation studies. The importance of RPDC loop parameter selection was pointed out, and it was noted that the integration loop of RPDC may be involved in system oscillations^13^. Some researchers investigated the enhancement of RPDC combined with VIC on the damping of doubly-fed motor oscillations^14^. However, the effect on the stability of small disturbances when the virtual inertia is increased is not discussed. According to some researchers, the optimal position and configuration amount of virtual inertia need to consider frequency stability and small signal stability^15^. In this paper, the role of RPDC and VIC in power system stability is investigated, and a procedure is used to calculate the optimal values of control parameters under the premise of ensuring system stability.

This paper first investigates the effect of damping and inertia of the dominant eigenvalues of small signal model. The damping and inertia values depend on the state of the control link in a DFIG, thus demonstrating the interaction between the damping and inertia performances and the system state. Based on these findings, updated rules that can improve the damping ratio of the grid are provided. The main idea is to use linearized summation methods to handle the sensitivity of dynamic eigenvalues of a nonlinear system to achieve an optimal system state equilibrium. The influence of variations in the parameters involved in the oscillation mode damping ratio on the sensitivity of the damping ratio is analyzed parametrically. Finally, by solving the proposed optimal configuration model of inertia and damping parameters, the optimal configuration scheme is obtained. The feasibility of the proposed optimization method is verified by simulation.

## Dynamic modeling

This section first explains the control model and its use including DFIG. Following that, the small-signal stability analysis methodology is presented.

### Model of virtual inertial and reactive power damping control

**Figure 1:**
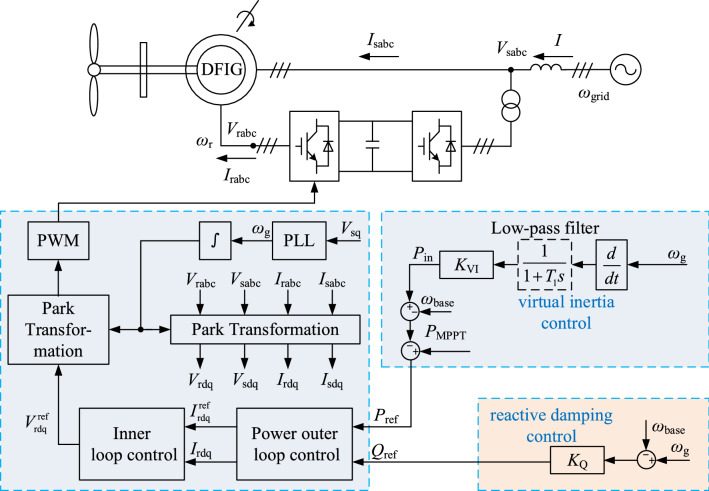
DFIG grid-connected system with damping and virtual inertia control system.

A DFIG with virtual inertia control can provide a connected system with additional virtual inertia. Also, the damping characteristics of a synchronous machine can be simulated using reactive damping control. The control diagram is shown in Fig. 1.

A crucial component of DFIG grid-connected systems is multi-timescale control. The dynamic properties of the storage element (rotor) at this time scale may be studied using the rotor speed time scale. The analysis of the virtual inertia response using rotor dynamic analysis is suited for the rotor speed time scale, which can be utilized to analyze the dynamic properties of the energy storage element (rotor) at this time scale. The analysis of the virtual inertia response characteristics utilizing rotor dynamics is suited for the rotor speed time scale, which can be utilized to analyze the dynamic features of the energy storage element (rotor) at this time scale. As a result, in order to give a clearer and more specific solution to the issue of DFIG equivalent inertia assessment, this study A simplified model of the DFIG is built for the rotor speed time scale, and the following assumptions are established and the following assumptions are made.

1. Ignoring the dynamic changes in the magnetic chain and the stator resistance losses. Ignoring the variations in wind speed and mechanical torque.
2. Regulation of AC current control is ignored. The output current vectors of the RSC and GSC can track their reference values instantly
3. The net-side converter is less dynamic compared to the rotor-side converter, so the net-side converter dynamics are ignored.

**Figure 2:**
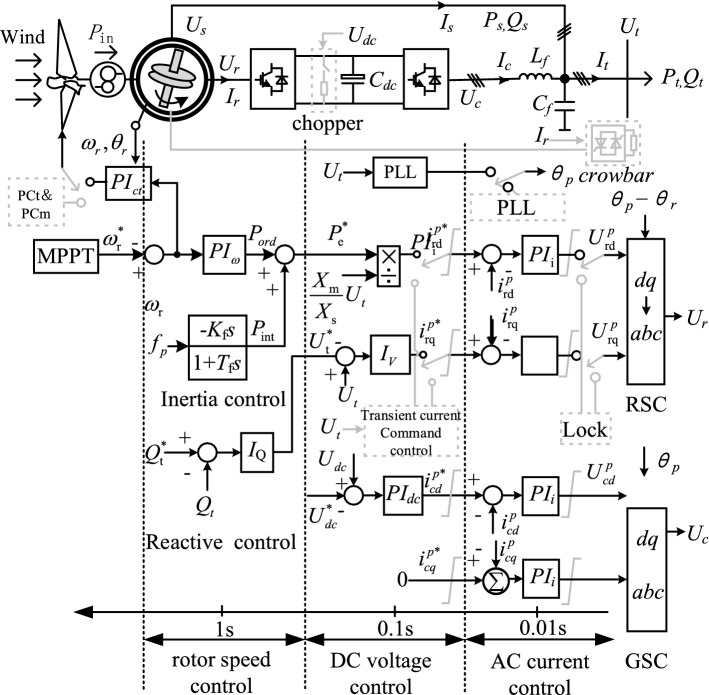
Typical control strategy and multi-scale division for doubly-fed wind turbines.

Figure 2 illustrates a typical vector control strategy for a doubly-fed wind turbine based on end-voltage orientation. There are various forms and capacities of energy storage elements within the doubly-fed wind turbine, such as mechanical rotors, DC capacitors and AC inductors. For each energy storage element, conventional and transient control are set up for different purposes. In order to maintain a stable state of each type of energy storage element, conventional controls such as rotor speed control, DC voltage control and AC current control are designed respectively; in order to protect each type of energy storage element from its own stress, corresponding transient controls and hardware circuit protection are designed respectively, such as emergency pitch control/brake device, transient current command control, compensation control, etc. shown in grey dashed lines. The DFIG's output power varies with grid disturbance. According to storage capacity, each energy storage element's state changes. Depending on storage capacity, each energy storage element's state varies, driving each conventional/transient controller to act or process is multi-scale and sequential.

In this scenario, conventional control is employed for shallow faults and deeper faults. When the fault is shallow, conventional control is utilized; otherwise, transient control is used. Paper^16^ describes the reaction of a doubly-fed turbine in various modes. This work focuses on the control time scale of a doubly-fed wind turbine's rotor speed when the fault is shallow, i.e. when conventional control mode is applied. Modeling/analysis. Inertia dominates this scale's reaction. Speed, inertia, and reactive power management dominate at this size^17^. In addition, this paper only focuses on the occurrence of three symmetrical fault events in the grid, the negative sequence control under asymmetrical faults is not considered for the time being.

**Figure 3:**
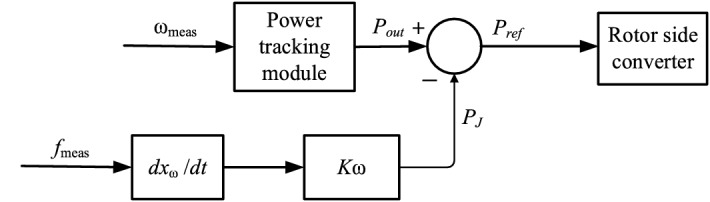
Virtual inertia control link block diagram.

The doubly-fed turbine is continuously operated in the maximum power point tracking (MPPT) mode due to the control of the rotor-side inverter. The commonly used virtual inertia control is an inertia model based on the frequency feedback on the wind power generation in full power mode used to provide an inertial response to the wind turbine and adjust the wind power's active output^18^. This control model is shown in Fig. 3.

The control link in Fig. 2 can be described by the following equation:

1 $$\dot{x}_{w} = \frac{1}{{T_{w} }}\left( {f_{meas } - x_{w} } \right)$$

After the virtual inertia control loop is introduced, the active power input of the DFIG rotor-side inverter is given by:

2 $$P_{{{\text{ref}}}} = P_{{{\text{opt}}}} - P_{J} = P_{{{\text{opt}}}} - K_{\omega } \dot{x}_{\omega }$$

where the introduced intermediate variable is $\dot{x}_{\omega }$; *T*_*ω*_ is the time constant; active power in the MPPT mode is *P*_opt_ ; output power of the virtual inertia control link is *P*_J_; virtual inertia control link scale factor is *K*_ω_, and it has a constant positive value.

To facilitate the analysis, the differential loop of the virtual inertia control link is simplified to a purely differential link^19^, and the active reference can be expressed as follows:

3 $$P_{{\text{ref }}} = P_{{\text{opt }}} - K_{\omega } \dot{f}_{{\text{meas }}}$$

where the virtual inertia of a DFIG is provided by the virtual control link; the measured angular frequency *f*_meas_ = ω_meas_/(2π); The equivalent inertia of the virtual inertia control link to the power system is

4 $$2H_{\omega } \dot{\omega }_{{\text{meas }}} = P_{{{\text{opt}}}} - P_{{\text{s}}}$$

5 $$H_{\omega } = \frac{{K_{\omega } }}{4\pi }$$

With the introduction of reactive power damping control, the reactive power generated by the control loop is given by^20^:

6 $$K_{{\text{d}}} \left( {\omega_{g} - \omega_{{\text{base }}} } \right) = Q_{{\text{ref }}} - Q_{{{\text{base}}}} = Q_{{\text{ref }}}$$

where *K*_d_ is the damping factor of the reactive damping control link; *Q*_base_ is the reactive power compensation generated by the system, and normally it has a value of zero.

Thus, doubly-fed motors with virtual inertia control and reactive damping control can provide virtual inertia and damping support to the grid. However, during electromechanical transients, the control loop may cause low-frequency oscillations due to the coupling of the doubly-fed motor with other synchronous generators.

### Model of power systems including DFIG

In the power system analysis, it is considered that multiple DFIG wind turbines form a wind farm. The model is mainly used for small signal analysis of multi-generator power systems. In power systems, the synchronous generator (SG) is represented by a third-order model, where the excitation power unit is represented by a first-order model^21^. Assuming that all turbines in a wind farm have the same type and operating conditions, turbine parameters can be approximated to the calculated parameters of a single turbine^22^. Thus, the equivalent model of a wind farm can be described by the set of dynamic characteristic equations of a DFIG, while the doubly-fed generator model is shown in the Appendix. Its state variable ***x*** is ***x*** = [*β*_ref_ *ω*_r_ *ω*_gen_ *θ*_s_ *i*_sd_ *i*_sq_ *E*_d_ *E*_q_ *u*_dc_ *x*_1_ *x*_2_ *x*_3_ *x*_4_ *x*_5_ *x*_6_ *x*_7_ *x*_8_ *δ*_*i*_* ω*_*i*_* E*_*fi*_* E´* i ], where *δ*_*i*_* ω*_*i*_* E*_*fi*_* E´* i and are the first work angle, speed, excitation potential, and transient potential of the *i*_*th*_ synchronous generator. The state variables that are often of interest when exploring how inertia control changes the coupling between the dynamic characteristics of a doubly-fed wind turbine and the system are as follows:

7 $$y = \left[ {\delta_{1} ,\omega_{1} ,I_{{{\text{dr}}}} ,I_{{\text{d ref}}} ,x_{{{\text{PLL}}}} , \, I_{{{\text{qr}}}} ,I_{{\text{q ref}}} ,U_{{{\text{dr}}}} ,s,\theta_{{{\text{shaft}}}} ,\omega_{{{\text{tur}}}} } \right]$$

where *δ*_1_ and *ω*_1_ are the work angle and speed of the synchronous generator, respectively; *s*, θ_shaft_ and *ω*_tur_ are the state variables of the mechanical loop of the doubly-fed wind turbine; the remaining variables denote the state variables of the induction motor and control link of the doubly-fed wind turbine. Near the equilibrium point, the differential equations of these state variables are linearized to get the characteristic matrix equation for the small-signal stability analysis of a power system with a wind farm that gets power from two different sources.

8 $$\frac{{{\text{d}}\Delta {\mathbf{x}}}}{{{\text{dt}}}} = {\mathbf{A}}\Delta {\mathbf{x}}$$

The relevant part of the system characteristic equation can represent the coupling relationship between the DFIG and SG dynamic characteristics.

9 $$\frac{d\Delta x}{{dt}} = \left[ {\begin{array}{*{20}l} {A_{11} } \hfill & {A_{12} } \hfill & {A_{13} } \hfill \\ {A_{21} } \hfill & {A_{22} } \hfill & {A_{23} } \hfill \\ {A_{31} } \hfill & {A_{32} } \hfill & {A_{33} } \hfill \\ \end{array} } \right]\Delta x$$

where A_11_, A_22_, and A_33_ describe the dynamic characteristics of the SG, DFIG, and the mechanical part of the wind turbine, respectively; the other variables indicate the characteristics of the coupling of the parts with other different dynamic components.

Equation (11) can also be expressed as a state equation consisting of the state variables of the synchronous generator SG, the DFIG of the doubly-fed turbine and the mechanical part of the DFIG.

10 $$\left[ {\begin{array}{*{20}c} {\Delta {\dot{\mathbf{x}}}_{{{\text{SG}}}} } \\ {\Delta {\dot{\mathbf{x}}}_{{{\text{DFIG}}}} } \\ {\Delta {\dot{\mathbf{x}}}_{{{\text{MECH}}}} } \\ \end{array} } \right] \, = {\mathbf{A}}_{s} \left[ {\begin{array}{*{20}c} {\Delta {\mathbf{x}}_{{{\text{SG}}}} } \\ {\Delta {\mathbf{x}}_{{{\text{DFIG}}}} } \\ {\Delta {\mathbf{x}}_{{{\text{MECH}}}} } \\ \end{array} } \right] = \left[ {\begin{array}{*{20}l} {A_{{{11}}} } \hfill & {A_{{{12}}} } \hfill & {A_{{{13}}} } \hfill \\ {A_{{{21}}} } \hfill & {A_{{{22}}} } \hfill & {A_{{{23}}} } \hfill \\ {A_{{{31}}} } \hfill & {A_{{{32}}} } \hfill & {A_{{{33}}} } \hfill \\ \end{array} } \right]\left[ {\begin{array}{*{20}c} {\Delta {\mathbf{x}}_{{{\text{SG}}}} } \\ {\Delta {\mathbf{x}}_{{{\text{DFIG}}}} } \\ {\Delta {\mathbf{x}}_{{{\text{MECH}}}} } \\ \end{array} } \right]$$

where *A*_12_ and *A*_13_ relate to the system tidal equation; they contain non-zero elements regardless of whether the equivalent load has dynamic characteristics^23^.

## Small-signal stability analysis

In this section, the conventional small-signal DFIG model and the small-signal model with virtual inertial and reactive damping control loops are presented. The expressions of the eigenvalues and damping ratios of the system under the action of damped and inertial control loops are derived based on the simple model of the two devices. In addition, an attempt is made to reveal the influence of the inertia and damping control loops on the small-signal stability of the system using the eigenvalue resolution method^24^. Finally, the eigenvalue analysis method commonly used to analyze the stability of small signals and their parameters, including the equation of state, eigenvalues, and participation factors of the components corresponding to eigenvalues, is introduced.

### Small-signal model of control loop

In the conventional DFIG control loop section, the time constant of the control link is much smaller than that of the mechanical section. The time constants of transient processes dominated by rotor currents are much smaller than the mechanical time constants^15^. Therefore, the dynamic process defined by Eqs. (1)–(8) is much faster than the dynamic process expressed by Eqs. (9)–(12). Thus, the steady-state solution to the dynamic process represented by Eqs. (1)–(8) is given by:

11 $$- \frac{{X_{{\text{m}}} }}{{X_{{\text{s}}} }}U_{{\text{t}}} I_{{q{\text{r}}}} = P_{{{\text{sref}}}}$$

where *X*_m_ is the mutual stator-rotor inductance; *X*_s_ is the stator reactance; Ut is the stator voltage; Iqr is the rotor q-axis current; *P*_sref_ is the active power.

In the maximum wind power tracking mode, the speed control module can rapidly provide a reasonable power output *P*_sref_ based on the current wind speed and wind turbine speed. The total output power of DFIG is balanced with the wind power. According to Eq. (9), when there is an outward disturbance, the control link of wind turbines can rapidly adjust *I*_qr_ to balance the system inertia, which makes the dynamic mechanical characteristics of the DFIG decoupled from the system.

According to Eqs. (A9)–(A12) in appendix, only the rotor side current and the corresponding control parameter changes affect the damping parameters. This indicates that the additional damping control method based on the rotor-side current loop exhibits decoupling characteristics for other system parameter changes.

After introducing the virtual inertia control link and reactive damping control, the expanded state variables are expressed as follows:

12 $$y = \left[ {\delta_{{1}} ,\omega_{{1}} ,I_{{{\text{dr}}}} ,I_{{\text{d ref}}} ,{\text{ x}}_{{{\text{PLL}}}} ,{\text{ I}}_{{{\text{qr}}}} ,I_{{\text{q ref}}} ,{\text{ X}}_{\omega } ,{\text{ X}}_{D} ,U_{{{\text{dr}}}} ,s,\theta_{{{\text{shaft}}}} ,\omega_{{{\text{tur}}}} } \right]$$

The previous section analyzes the coupling characteristics of variables based on time constants of oscillatory modes. On the time scale, the rotor-side voltage has a high response rate. It is relatively decoupled from the other state variables and thus does not affect the small disturbance stability of the system^25^. Since the time constants of the inertia and damping controls are close to those of the transient electromechanical oscillation process, the control links of virtual inertia and system damping are the main factors affecting the small disturbance stability of doubly-fed generators.

This paper studies only the small-signal stability for small disturbances of inertia and damping. Therefore, parameters unrelated to the small-signal stability can be omitted or simplified. Thus, only the critical system nodes containing system parameters are analyzed, while nodes that do not have damping or inertia are removed from the model, so the model may not maintain the original network. Next, a small disturbance model containing dynamic system parameters is constructed.

**Figure 4:**
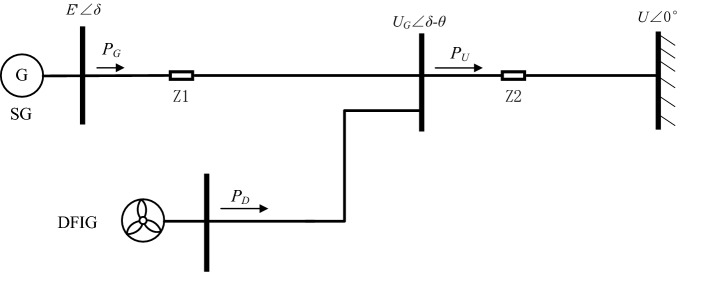
Diagram of two areas power system.

For a doubly-fed wind turbine and a synchronous generator in the system, the network between them can be approximated to the components connected to the network, forming the system shown in Fig. 4.

where PG and PD are the active power transmitted via the contact line through SG and DFIG. E'∠δ is the instantaneous voltage of SG. UG is the voltage value at the contact. U is the voltage on the busbar of other equipment, set as the ideal condition. Z_1_ and Z_2_ are the line impedance parameters. δ and θ denote the phase angle difference from the power inputs SG and DFIG to the contact point voltage UG, respectively.

The DFIG is set as the equilibrium node at the access point with the grid, the generator adopts the classical model, and the mechanical power PG is constant. Then the equation of motion of the node can be written as:

13 $$\left\{ {\begin{array}{*{20}l} {H_{{\text{G}}} \frac{{{\text{d}}\omega_{{\text{s}}} }}{{{\text{dt}}}} = P_{m} - P_{G} - {\text{D}}\left( {\omega_{{\text{s}}} - \omega_{{\text{e}}} } \right)} \hfill \\ {\frac{{{\text{d}}\delta }}{{d{\text{t}}}} = \omega_{{\text{s}}} - \omega_{{\text{e}}} } \hfill \\ \end{array} } \right.$$

where HG, PG are the inertia and electromagnetic power of the synchronous generator, respectively; D is the damping factor.

The equation of perturbation motion of the equilibrium node is obtained by finding the amount of small perturbation to the above equation. The conventional unitary oscillation equation for SG can be expressed by^26^:

14 $$2H_{G} p^{2} {\Delta }\delta + D_{G} p{\Delta }\delta + {\Delta }P_{G} = 0$$

where *D*_*G*_ is the constant of inertia and damping; Δ*P*_*G*_ is the active power variation; Δ*δ* is the power angle variation of an SG; p is the differential operator. Let the active power P of the DFIG cause the phase angle change *θ* and the reactive power Q cause the system voltage amplitude change Δ*U*_*G*_. Then, each variable is set to its initial value, denoted by the subscript "0." Δ*P*_*G*_ and Δ*Q*_*G*_ can be expressed by:

15 $${\Delta }P_{G} = \frac{{E^{\prime}U_{G} }}{{Z_{1} }}\cos \theta_{0} + \frac{{E^{\prime}\sin \theta_{0} }}{{Z_{1} }}{\Delta }U_{G}$$

16 $$\begin{array}{*{20}c} {{\Delta }Q_{G} = \frac{{ - E^{\prime}U_{G0} }}{{Z_{1} }}\sin \theta_{0} {\Delta }\theta + \frac{{E^{\prime}\cos \theta_{0} - 2U_{G0} }}{{Z_{1} }}{\Delta }U_{G} } \\ { = {\Delta }Q_{\theta } + {\Delta }Q_{V} } \\ \end{array}$$

17 $$K_{G} = \frac{{E^{\prime } U_{G} }}{{Z_{1} }}\cos \theta_{0}$$

By substituting (17) and (18) into (15), the system equation is obtained as follows:

18 $$2H_{G} \Delta \ddot{\delta } + D\Delta \dot{\delta } + a\Delta \delta = 0$$

Let the time constant of a PLL be infinitesimal; then, the frequency obtained by the virtual inertia control loop in a DFIG is the system frequency. Assuming that the angular frequency variation is consistent in the system presented in Fig. 3, the active output power variation with virtual inertia can be obtained by:

19 $$\Delta P_{D} = - \frac{{K_{\omega } }}{2p}\Delta \omega_{c} = - \frac{{K_{\omega } }}{2p}\Delta \ddot{\delta }$$

where Δ*ω*_c_ is the measured angular frequency value. Since the reactive output affects only the voltage amplitude U at the common connection point, the reactive damping control loop can be used to denote *Q*_*G*_.

20 $$\Delta P_{D} = - \frac{{K_{\omega } }}{2p}\Delta \omega_{c} = - \frac{{K_{\omega } }}{2p}\Delta \ddot{\delta }$$

The value of the reactive power damping control output derived from Eq. (4) is given by:

21 $$\Delta Q_{WTG} = - K_{{\text{d}}} \Delta \omega = - K_{{\text{d}}} p\Delta \delta$$

Further, combining Eqs. (22) and (20), we have:

22 $$\Delta U_{G} = \frac{{Z_{1} K_{Q} }}{{2U_{G0} - E^{\prime } \cos \theta_{0} }}p\Delta \delta$$

The power variation Δ*P*_u_ at the connection point is expressed by:

23 $$\Delta P_{{\text{U}}} = K_{L} (\Delta \delta - \Delta \theta ) + \frac{{U\sin \left( {\delta_{0} - \theta_{0} } \right)}}{{Z_{2} }}\Delta U_{G}$$

24 $$K_{L} = \frac{{U_{G} U}}{{Z_{2} }}\cos \left( {\delta_{0} - \theta_{0} } \right)$$

By combining Eqs. (24)–(26), it can be obtained that:

25 $$a\Delta \theta = \left( {K_{\omega } \frac{{d^{2} \Delta \delta }}{{dt^{2} }} + K_{{1}} \frac{d\Delta \delta }{{dt}} + K_{L} \Delta \delta } \right)$$

where $$K_{1} = \left( {\frac{{U\sin \left( {\delta_{0} - \theta_{0} } \right)}}{{Z_{2} }} - \frac{{E^{\prime } \sin \theta_{0} }}{{Z_{1} }}} \right)\frac{{Z_{1} K_{d} }}{{2U_{G0} - E^{\prime } \cos \theta_{0} }}$$, $$a_{0} = K_{G} + K_{L}$$.

Further, by substituting (27) into (20) can be expressed as follows:

26 $$(2H_{G} + H_{DFIG} )p^{2} {\Delta }\ddot{\delta } + (D_{G} + D_{DFIG} )p{\Delta }\dot{\delta } + a_{1} {\Delta }\delta = 0$$

where $H_{DFIG} = \frac{{K_{\omega } a_{0} z_{2} }}{{2\pi U_{G} U\cos \left( {\delta_{0} - \theta_{0} } \right)}} > 0$, $a_{1} = \frac{{E^{\prime}U_{G} U\cos \theta_{0} \cos \left( {\delta_{0} - \theta_{0} } \right)}}{{E^{\prime}z_{2} \cos \theta_{0} + Uz_{1} \cos \left( {\delta_{0} - \theta_{0} } \right)}}$, $K_{QD}^{\prime } = \left( {\frac{{U\sin \left( {\delta_{0} - \theta_{0} } \right)}}{{Z_{2} }} + \frac{{K_{L} }}{{K_{G} }}\frac{{E^{\prime } \sin \theta_{0} }}{{Z_{1} }}} \right)\frac{{Z_{1} K_{Q} }}{{2U_{G0} - E^{\prime } \cos \theta_{0} }}$, $D_{DFIG} = K_{G} K_{QD}^{\prime } /a$. where *H*_DFIG_ and *D*_DFIG_ denote the equivalent inertia and damping coefficients provided by the control loop, respectively; *H*_DFIG_ is defined by virtual inertia control; *D*_DFIG_ is defined by reactive damping control. In short, the virtual inertia control enhances the equivalent inertia of the system.

### Eigenvalue analysis

Eigenvalue analysis is an efficient method to analyze the damping characteristics of a system and the oscillation modes present in the system based on eigenvalues of the state matrix of the system. Its mathematical basis is the Lyapunov linearization method. The differential equation consisting of state variables near the equilibrium point is linearized to obtain the eigenmatrix equation for small-signal stability analysis of a power system containing a doubly-fed wind farm^27^.

In This study, the effect of a wind turbine on the small disturbance stability of a system after the introduction of inertia is observed. Using the mode analysis method, different oscillation modes dominated by certain system components can be obtained. The participation factors and damping ratios of different state variables are derived according to the corresponding characteristic roots, which indicate the degree of participation of the state variables in different system components in the oscillation mode and the influence on the damping ratio. In addition, the degree of influence of a specific component on the small-signal stability of a system is expressed. The specific method and participation will be explained in detail in the next section.

When the wind turbine load changes, the output torque imbalance reduces the resonance frequency and may cause low frequency oscillations in the output power of the wind power system, thus affecting the safety and stability of the wind turbine and its system. Among the main oscillations caused are:

1. The DFIG unit internal shaft system oscillation model with an oscillation frequency of 2.18 and a relatively low damping ratio of 0.04.
2. The intra-regional oscillation containing units G3 and G4 with an oscillation frequency of 1.44 Hz and an oscillation damping ratio of 0.12.
3. The intra-regional oscillation model containing synchronous units G1 and G2. The oscillation frequency and damping ratio of this model are 1.25 and 0.06 respectively.
4. Inter-regional oscillation model dominated by the control link of the wind farm. In the control coefficient variation has a significant effect on the oscillation frequency.

For the system defined by (20), the dominant eigenvalues of the system and the corresponding damping ratios are respectively given by^28^:

27 $$\lambda_{i} = \left\{ {\begin{array}{*{20}l} { - \frac{a}{{d_{i} }}} \hfill & {{\text{ if }}m_{i} = 0} \hfill \\ {\frac{{ - d_{i} \pm \sqrt {d_{i}^{2} - 4m_{i} a} }}{{2m_{i} }}} \hfill & {{\text{ otherwise}}{. }} \hfill \\ \end{array} } \right.$$

28 $$\zeta \, = \frac{{d_{i} }}{2}\sqrt {\frac{1}{{m_{i} a}}}$$

where *m*_i_ is the inertia parameter in a system, *d*_i_ is the damping parameter, and a is the system electrical quantity parameter.

According to Eq. (30), the increase in inertia m causes the damping ratio of a system to decrease, which can affect the power angle stability of the system. An appropriate increase in the damping coefficient can improve the damping ratio of the system. It mainly affects the inter-regional oscillation mode, which indicates that grid-connected wind turbines cause a decrease in the small-signal stability of the system when virtual inertia control is used. To ensure the small-signal stability of the system, reactive damping control needs to be used in conjunction with virtual inertia control to achieve an optimal damping ratio of the system.

## Proposed optimization method

This section analyzes the effect of the doubly-fed wind turbine on system stability under small disturbances from the perspective of the system, considering the addition of inertia and reactive damping controls and how the added control loop changes the coupling relationship between the dynamic characteristics of the doubly-fed wind turbine and the system.

In small disturbance stability analysis, the system state-space equation has often been used. Still, the analytical expression of the mode damping ratio considering system parameters can be difficult to obtain by the state-space analysis. To solve this problem, a solution algorithm based on the sensitivity of the damping ratio to the system parameters is proposed in this paper. Since the DFIG incidental control loop parameters mainly affect the interval oscillation damping ratio, the main goal of optimization is to maximize the damping ratio for the worst case of interval oscillation, ensuring optimal attenuation in terms of system oscillations. It is assumed that both damping and inertia can be adjusted within constraints.

The damping ratio *ζ* is that it indicates the nonlinearity in sensitivity to the decision variables. The goal of an optimization algorithm is to find a local optimal solution by iteratively solving a linearized approximation. Specifically, the damping and inertial sensitivities of the damping ratio are used to compute a new damping ratio and sensitivity to the continuous parameter update of the damping ratio linearization in the next iteration, which it is necessary to have information on two parameters. First, calculating the sensitivity requires finding system eigenvalues, which must be recalculated at each iteration. In this study, an approximation of system eigenvalues is used. The second concern relates to the iteration approach. In this study, an iterative approach is adopted, fixing the step size of each parameter. The values of damping ratios are compared after the first and second iterations. The iterative algorithm stops if the new value is worse than the previous value or if the difference between them is less than the predefined constraint value. Although this approach has been proven to be useful in improving the stability of simple networks, employing more powerful solution strategies for complex systems might be necessary.

The superscripts "0," "*ν*," and "*ν* + 1" correspond to the first, current, and next iterations, respectively. The difference between the current and next iterations is expressed by:

29 $$\left\{ \begin{gathered} \Delta d_{i}^{\nu + 1} = d_{i}^{\nu + 1} - d_{i}^{\nu } \hfill \\ \Delta m_{i}^{\nu + 1} = m_{i}^{\nu + 1} - m_{i}^{\nu } \hfill \\ \end{gathered} \right.$$

For the optimized cost function, the constraints| are expressed as follows.

30 $$\sum\limits_{{i \in {\mathcal{K}}}} {\left| {d_{i} } \right|} \, \le d^{{\text{sum }}}$$

31 $$\sum\limits_{{i \in {\mathcal{M}}}} {\left| {m_{i} } \right|} \, \le m^{{{\text{sum}}}}$$

32 $$d_{j}^{\min } \, \le d_{j}^{\nu + 1} \le d_{j}^{\max }$$

33 $$m_{j}^{\min } \, \le m_{j}^{\nu + 1} \le m_{j}^{\max }$$

34 $$\Delta d_{i}^{\min } \, \le \Delta d_{i}^{\nu + 1} \le \Delta d_{i}^{\max }$$

35 $$\Delta m_{i}^{\min } \, \le \Delta m_{i}^{\nu + 1} \le \Delta m_{i}^{\max }$$

It has been shown that the inertia m has different effects on the small disturbance stability and frequency stability. Increasing the value of m can improve the frequency stability of the system, but it can also deteriorate the small disturbance stability of the system. To ensure the system has good small disturbance stability and frequency stability at the same time, the value of m should be determined considering the constraints of the two types of stability simultaneously.

Constraints (32) and (33) limit the total amount of inertia and damping of the system in the optimization process, respectively; meanwhile, constraints (34) and (35) denote the boundaries of damping and inertia, respectively. Further, step size constraints (36) and (37) limit the variations of parameters in each iteration. Namely, the optimization algorithm cannot converge in a nonlinear domain without such a constraint. Then, the sensitivity optimization is given by:

36 $$\zeta_{i}^{\nu + 1} = \zeta_{i}^{\nu } + \sum\limits_{{j \in {\mathcal{K}}}} {\frac{{\partial \zeta_{i}^{\nu } }}{{\partial d_{j} }}} \Delta d_{j}^{\nu + 1} + \sum\limits_{{j \in {\mathcal{M}}}} {\frac{{\partial \zeta_{i}^{\nu } }}{{\partial m_{j} }}} \Delta m_{j}^{\nu + 1}$$

Equation (38) calculates a new damping ratio for the oscillatory mode using the damping ratio cumulative for the control parameters k and m sensitivity. The superscript *v* denotes the number of iterations and will not be described separately in this paper.

The optimization goal is to maximize the objective function under defined constraints. The termination condition is set to check whether the damping result obtained in two consecutive iterations is less than an arbitrary constant defined according to the accuracy requirement. In addition, if the number of iterations reaches the upper limit, the calculation is also terminated.

**Figure 5:**
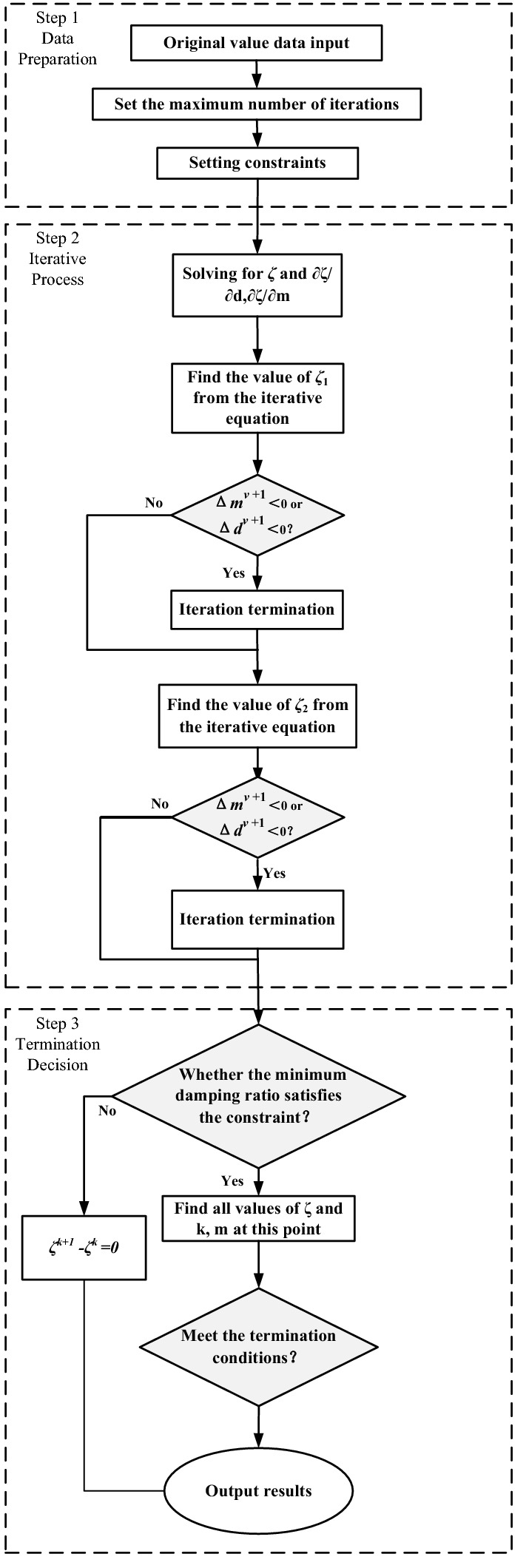
Diagram of two areas power system.

Based on the sensitivity of the damping ratio to the variable parameters, the algorithm used to solve the optimization model (31)–(37) is proposed, and its flowchart is presented in Fig. 5. This algorithm includes three main steps constraint setting, iterative solution determination, and termination condition definition, which are described in detail in the following.

(1) *Step1* Data preparation.

It is necessary to determine conditions that satisfy the minimum inertia required by the system and the maximum inertia provided by the virtual inertia control link according to constraints (32)–(37). Then, the number of iterations is set to zero, and the initial values k0 and m0 are assigned to T(0); T(k) denotes the parameter of the *m*th wind farm at the kth iteration.

(2) *Step2* Iterative solution.

In this phase, *ζ* is first solved for with according to Eq. (28). As explained previously, the algorithm is used to determine the direction of optimization through sensitivity. In the (*k* + 1)st iteration, after the *m*th wind farm *ζ* needs to be determined whether it is less than zero, and if it is less than zero then it is set to zero. After the NDFIG wind farms have been obtained, it is judged whether the constraints are satisfied and if so, *ζ*(v) is updated to *ζ*(k + 1); if not, the *k*th result is retained.

(3) *Step3* Termination decision.

Since the optimization objective is to maximize the damping ratio of the interval oscillation mode, then the termination condition can be set to be less than any constant chosen according to the accuracy requirements. In addition, if the number of iterations reaches the upper limit, the calculation is also terminated.

## Parameter analysis and Simulation implementation

### Introduction to simulation systems

**Figure 6:**
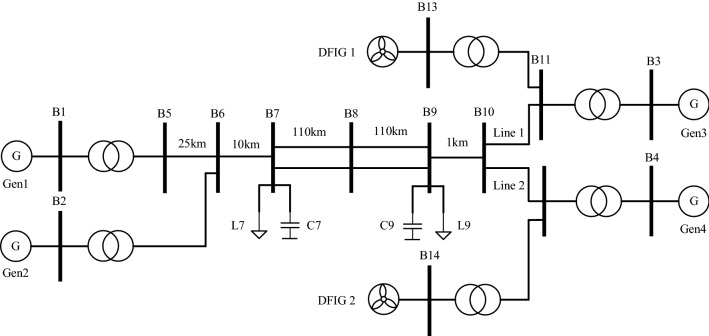
Simulation diagram of a four-generator dual-zone system.

In this paper, the simulation system shown in Fig. 6 is built in DIgSILENT/PowerFactory. The diagram shows that the system is divided into two areas connected by transmission lines, with the left side as the sending end and the right side as the receiving end. Gen1 and Gen2 are synchronous generators on the sending side, and Gen2 is the reference motor. On the other hand, there are two wind farms, Wind Farm 1 and Wind Farm 2, on the receiving side. These farms have turbines fed from both sides and two synchronous generators, Gen3 and Gen4, which have a power output of 900 MW on the transmitter side. A study of how wind power with virtual inertia control affects the system performance in an interconnected grid found that when wind farms with virtual inertia control are on the transmitter side, they can make the oscillation pattern between regions unstable. According to the paper^29^, when the wind farm with virtual inertia control is at the sending end, the oscillation pattern between regions will be unstable. The theoretical derivation of the paper shows that the suppression of system oscillations is better when the virtual inertia is assigned far from the center of inertia or in the low inertia region. In the Appendix, the generator and excitation parameters, as well as the initial control parameters of the control system discussed in this paper, are given.

The frequency stability of the power system is related to the unit inertia^30^. To ensure frequency stability, the minimum total inertia of the system should satisfy Eq. (38).

37 $$H_{{\text{t}}} = \frac{{\sum\limits_{n = 1}^{{N_{{{\text{SG}}}} }} {H_{{{\text{SG}},n}} S_{{{\text{SG}},n}} } + \sum\limits_{m = 1}^{{N_{{{\text{DFIG}}}} }} {H_{\omega ,m} S_{{{\text{DFIG}},m}} } }}{{\sum\limits_{n = 1}^{{N_{{{\text{SG}}}} }} {S_{{{\text{SG}},n}} } + \sum\limits_{m = 1}^{{N_{{{\text{DFG}}}} }} {S_{{{\text{DFIG}},m}} } }}$$

where *H*_t_ is the overall inertia of the system; *H*_SG,n_ is the time inertia constant of the nth S_G_; *H*_ω,m_ is the equivalent inertia of the *m*th doubly-fed wind turbine; *S*_SG_ and *S*_DFIG_ are the equivalent inertia values of the synchronous generator and doubly-fed wind turbine, respectively; *H*_*ω,m*_ is the equivalent inertia of the *m*th doubly-fed turbine; SSG and S_DFIG_ are the nominal capacities of the SG and DFIG, respectively; *N*_SG_ and *N*_DFIG_ are the nominal capacities of the synchronous generator and doubly-fed turbine, respectively; *H*_min_ indicates the minimum inertia value for stable operation of the system, and it is expressed as follows :

38 $$\begin{gathered} \sum\limits_{m = 1}^{{N_{{\text{DFG }}} }} {K_{\omega ,m} } S_{{{\text{DFIG }},m}} \ge 4\pi \hfill \\ \, \left( {H_{\min } \left( {\sum\limits_{n = 1}^{{N_{{{\text{SG}}}} }} {S_{{{\text{SG}},n}} } + \sum\limits_{m = 1}^{{N_{{{\text{DFIG}}}} }} {S_{{{\text{DFIG}},m}} } } \right) - \sum\limits_{n = 1}^{{N_{{{\text{SG}}}} }} {H_{{{\text{SG}} ,n}} } S_{{{\text{SG}} ,n}} } \right) \hfill \\ \end{gathered}$$

This example's investigation starts with a sensitivity analysis to determine the impact of virtual inertia. The study first checks, through sensitivity analysis, the influence of the virtual inertia distribution on the interval oscillation pattern, and then validates the correctness of the suggested model and method. The study of this case first validates, via sensitivity analysis, the influence of virtual inertia allocation on the interval oscillation mode, and then checks the efficacy of the suggested model and method.

### Effect of parameter assignment on interval oscillation patterns

Let *I*_1_ and *I*_2_ be the sensitivity values of interval oscillation damping ratio *ζ* to inertia as *m*_1_ in area 1 and inertia as *m*_2_ in area 2. Combining pattern frequencies and participation factors, the interval oscillation modes of the system can be obtained. The sensitivity analysis enables a more intuitive insight into the effect of the virtual inertia m and the damping parameter d on the damping ratio *ζ* of the interval oscillation mode. There are 2 main factors that influence the distribution of virtual inertia. One is the electrical distance of the virtual inertia from the load and the other is the capacity of the wind farm where the virtual inertia control link is added. In this paper, we first consider the influence of inertia and the electrical distance from the load center on the sensitivity results. *I*_1_ and *I*_2_ are the sensitivities of the interval oscillation damping ratios for wind farm 1 and wind farm 2 respectively; the damping parameters are taken to be the same value. The capacities of wind farms 1 and 2 are the same, and *L*_1_ and *L*_2_ are the lengths of lines 1 and 2. Combining the mode frequency with the participation factor, the ratio of the inter-zone oscillation mode of the system can be obtained as follows.

As can be seen from Table 1, when two wind farms have the same capacity, keeping L_2_ length at 10 km, *I*_1_: *I*_2_ increases as *L*_1_ grows. Adding virtual inertia to wind farm 1 improves the inter-area oscillation mode damping ratio. Since the lengths of *L*_1_ and *L*_2_ measure the electrical distance from the wind farm to the load node, it can be seen that in this example, assigning the virtual inertia to a wind farm that is further away from the load electrical distance is more likely to The damping ratio of the grid interval oscillation mode is improved. When the line length *L*_1_ is greater than *L*_2_, the damping ratios of the interval oscillation modes of the two wind farms are related to *I*_1_ and *I*_2_ as follows: where the solid line indicates the damping ratio represent *I*_1_. The dashed line shows the damping ratio represent *I*_2_.

**Table 1:** Relation between electrical distance and inertia distribution.

Case	Area1(MWA)	Area2(MWA)	*L*_1_ (km)	*L*_2_ (km)	*I*_1_/*I*_2_
Case 1	450	450	10	10	1
Case 2	450	450	20	10	1.487
Case 3	450	450	30	10	3.521
Case 4	450	450	10	5	0.625

It can therefore be concluded that in this example system, the allocation of virtual inertia to wind farms with a long electrical distance from the load or a large capacity results in a better damping ratio for the inter-area oscillation modes and is more conducive to small disturbance stability of the system.

This section demonstrates the influence of wind farm capacity on the sensitivity results, proving that the main factor influencing the values of the wind turbine control parameters *d* and *m* is the wind farm capacity. Only one interval oscillation mode exists in this example system. The results of the sensitivity analysis are shown in Table 2.

**Table 2:** Load data for wind turbines in the dual regional system grid.

Case	Area1(MWA)	Area2(MWA)	*d*_1_/*d*_2_	*I*_1_/*I*_2_
Case 1	450	450	3:1	3.0785
Case 2	450	450	2:1	2.0337
Case 3	450	450	1:1	1.0000
Case 4	500	400	1:1	1.7509
Case 5	600	300	1:1	3.2831
Case 6	600	300	1:2	2.1934

When two wind farms have the same capacity, *I*_1_ and *I*_2_ increase with the growth of transmission lines L_1_ and L_2_, which indicates that adding virtual inertia to wind farm 1 will improve the inter-area oscillation mode damping ratio. In this case, the virtual inertia is assigned to wind farms that are further away from the load electrically, which improves the grid's inter-area oscillation mode damping ratio. In this paper, an optimal turbine configuration location in the system is selected directly to exclude the influence of the configuration of virtual inertia devices of the system on the small disturbances analysis.

Under the premise of guaranteeing the maximum virtual inertia of system capacity provided by the equipment in the system, as constrained by Eq. (39), the total capacity of the two wind farms is kept constant. The capacity ratio of the two wind farms is continuously changed; namely, the capacity of wind farm 2 is reduced, while the capacity of wind farm 1 is increased. In addition, the sensitivity ratio *I*_1_:* I*_2_ increases with the capacity share of wind farm 1. Thus, in this case, the degree of influence of virtual inertia on system stability under small disturbances is positively related to the capacity share of wind farm 1. Hence, virtual inertia to wind farms with a larger capacity can increase the inter-area oscillation damping rate.

**Figure 7:**
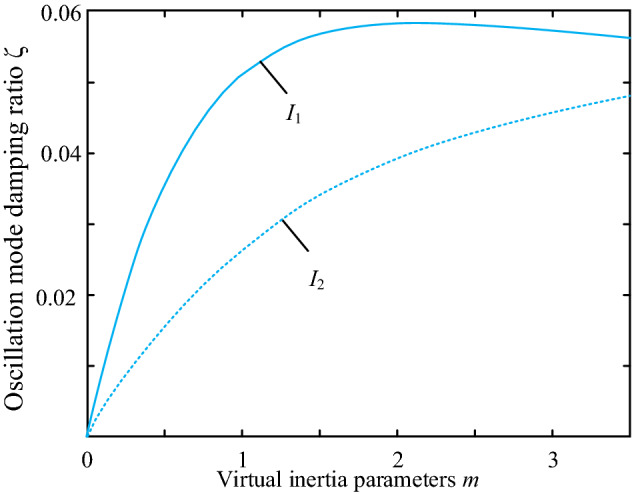
Comparison of sensitivity of oscillation mode damping ratio to damping coefficient.

Next we analyze the sensitivity of the damping *ζ* ratio to the fan damping control parameter *K*. The sensitivity equation is given by (21). Using the capacity parameters of the DFIG unit in Table 1 Case 4, the values of the sensitivity for each of the two regions are found, as shown in Fig. 7.

**Figure 8:**
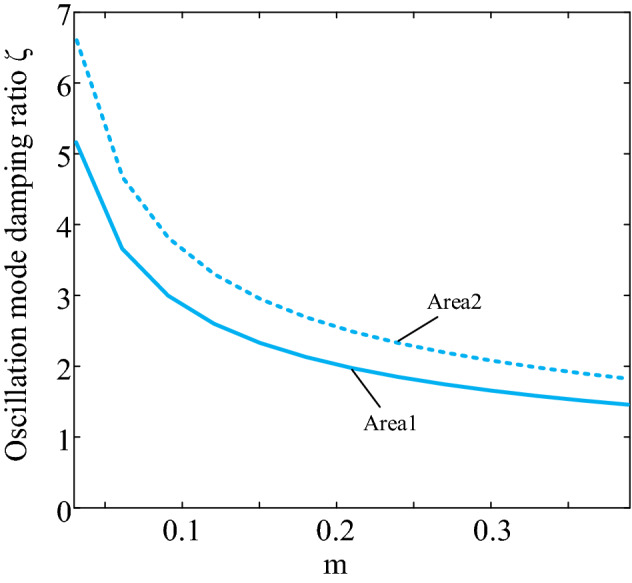
Comparison of sensitivity of oscillation mode damping ratio to damping coefficient.

According to Fig. 8, the sensitivity value of the oscillatory mode damping ratio to the damping factor of the system depends mainly on the magnitude of the inertia value. It indicates that the inertia value influences the configuration of the DFIG damping parameters in the system and is negatively related to the virtual inertia provided by DFIG. Also, Fig. 7 shows that the initial sensitivity of a region with a small capacity is considerable. Still, the sensitivity decreases fast with the virtual inertia, and the sensitivity of both areas tends to be the same. This indicates that the increasing effect of inertia on the damping ratio reaches a threshold value when the inertia increases to a particular matter.

Next, we utilize the characteristics listed in Table 1 for Case 4 to calculate how sensitive the damping ratio is to the magnitude of inertia. Figure 8 displays the outcomes using the derivative of the damping ratio with respect to the damping factor *d* as the vertical axis and the inertia control factor m as the horizontal axis.

**Figure 9:**
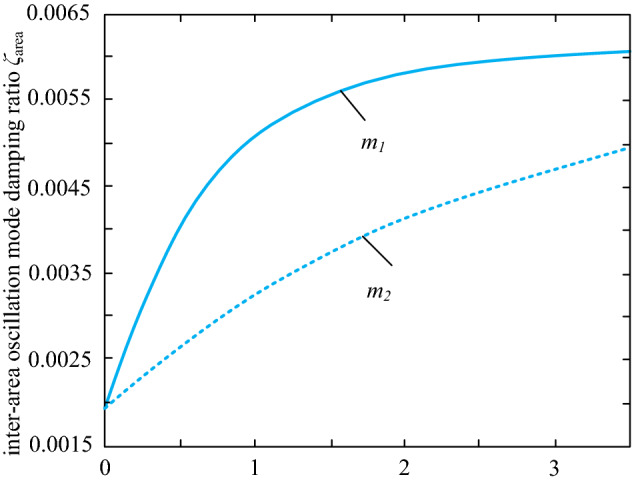
Changing trends of inter-area oscillation.

The solid line in Fig. 9 shows the sensitivity of the damping ratio to the virtual inertia in region 1, and the dashed line indicates the sensitivity of the damping ratio to the virtual inertia in region 2. According to the results in Fig. 8, the sensitivity values in both regions increase with inertia. However, the increase in the sensitivity value is more pronounced when increasing the virtual inertia in region 1. It can therefore be seen that in this example, when the damping coefficients are the same in both regions, the degree of influence of the virtual control coefficient on the stability of the system with small disturbances is positively related to the capacity share of wind farm 1, i.e. assigning the virtual inertia to a wind farm with a larger capacity in this example will improve the mode damping ratio.

### Program implementation and results analysis

According to the previous conclusions, the sensitivity of the damping ratio to the damping factor d depends mainly on the inertia value in a specific region of a system. In this paper, we selected the data of case 4 (Table 1) and the optimization algorithm (37) mentioned earlier is employed to determine an optimal damping ratio of the system and find the values of inertia and damping coefficient corresponding to optimal damping ratio. In the considered case, the minimum inertia of the system required for frequency stabilization is set to *H*_min_ = 5.12 s, and the following constraint can be obtained based on Eq. (40):

39 $$H_{\omega ,1} S_{1} + H_{\omega ,2} S_{2} \ge 382.71$$

Using the data of Case 4 (Table 1) converted according to the maximum virtual inertia output power provided by different capacities, it is set that *m*_1_ = 0.15, *m*_2_ = 0.25, *d*_1_ = 0.92, and *d*_2_ = 0.92. In the algorithm presented in Section III, the optimization step and termination condition are set to *u* = 0.1 and Δ*ζ* ≤ 1 × 10^−8^, respectively. The initial values and constraints are brought into the program for calculation and the optimization ends when 10 calculations are run and *ζ* reaches its maximum value.

**Figure 10:**
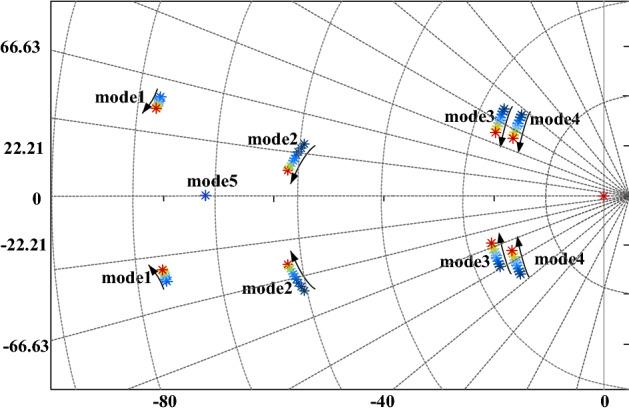
Comparison of eigenvalues after damping ratio optimization.

The surface shown in Fig. 10 illustrates the relationship between the damping ratio of the interval oscillation mode and the control parameters *m* and *d*. The scatter trajectory in the figure represents the optimization iteration process. The results show that the critical eigenvalues of the optimized interval oscillation mode are shifted to the left in the real part and to the horizontal axis in the imaginary part, and the small disturbance stability of the system is improved. The correctness of the proposed algorithm is verified.

**Figure 11:**
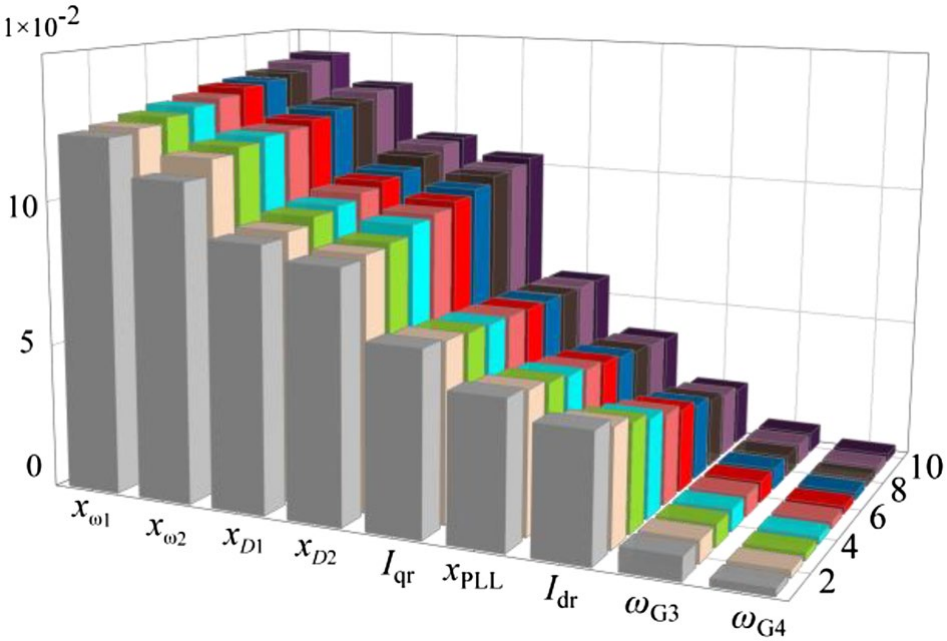
Participation factors for state variables.

In the following, we will derive the relationship between the state variable participation coefficient of the system control loop and the damping ratio of the inter-area oscillations from the change in the eigenvalues derived in Fig. 9, where we derive the change in the participation coefficient. The corresponding characteristic roots of the inter-area oscillation pattern in Fig. 10 are selected and ranked according to the magnitude of the state variable participation coefficients. As the system parameters change over the course of the iteration, the state variable participation factors change accordingly. The results obtained are shown in Fig. 11.

As shown in Fig. 11 the state variables of the control parameters dominate the oscillation mode. The virtual inertia control state variables *x*_*ω*1_ and *x*_*ω*2_ increase gradually with the parameters' values. The reactive damping control state variables participation factors *x*_*D*1_ and *x*_*D*2_ have the same changing. This is consistent with the previous conclusion that the inertia parameter assignment dominates the damping ratio. As the parameters increase, the damping ratio of the system increases, indicating that the system stability under small disturbances is improved.

**Figure 12:**
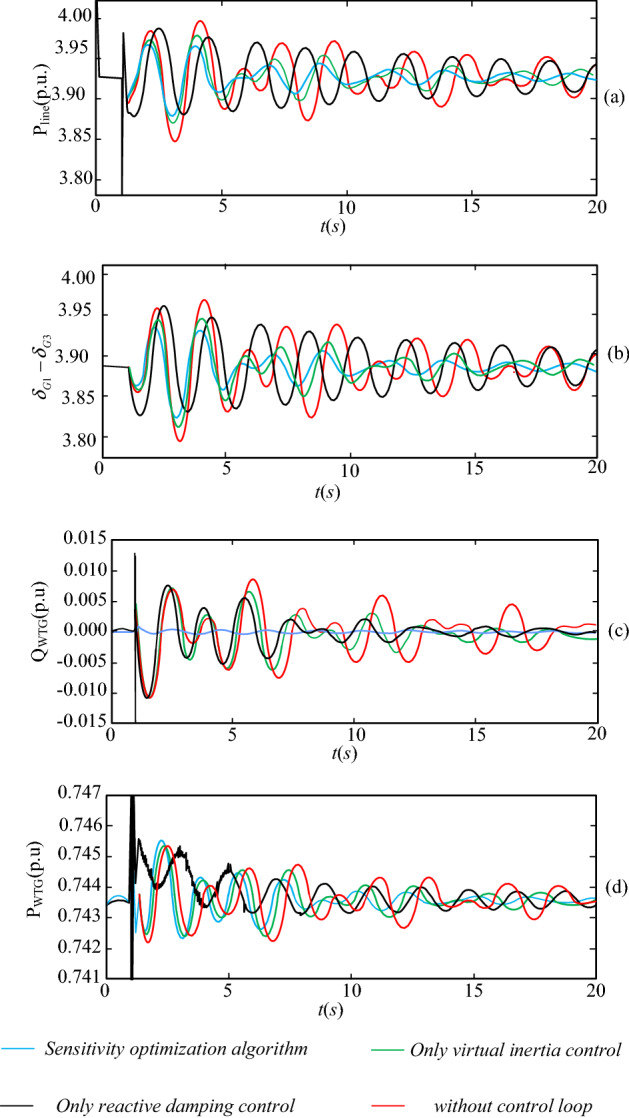
Power across the contact line after a fault.

The inter-area oscillation mode can be reflected in the dynamic response of the active power of the transmission line^31^. The amplitude of the oscillations reflects the small disturbance stability of the system. Due to virtual inertia control and reactive damping control, a doubly-fed generator is like to the synchronous generator in terms of inertia and damping characteristics. Namely, it can support the active system power for a short time after system power fluctuations. Assume that a three-phase short-circuit fault occurs at node B8 at t = 1 s, and the fault is addressed after 0.1 s. Then, power fluctuations at the contact line before and after optimization are as shown in Fig. 12.

The curves in these plots show that the system amplitude and oscillation times become significantly larger without the addition of reactive control and virtual inertial control to the DFIG. The parameters of the virtual inertia control and reactive power damping control are set separately and compared with the parameters derived in “Parameter analysis and Simulation implementation” section that include both controls. When no auxiliary control measures are introduced into the system, the system oscillates and tends to destabilize due to an inadequate damping ratio after a three-phase fault has occurred. The system stabilizes only after several cycles when other stabilization measures have taken effect. The WTGs, with the introduction of virtual inertia in the region, reduce the active output during this period, reducing the amount of active power mismatch in the system during the transient process. Of the four cases, the values of the two control parameters derived using the method of sensitivity in this paper give the best results in terms of oscillation amplitude and oscillation time after fault occurrence. It also allows the reactive and active power of the doubly-fed wind turbine to maintain its rated output value to the maximum extent possible in the event of a fault. Simulation results show that the optimization method mentioned in this paper performs best in terms of oscillation amplitude and oscillation time of the transmission line, and the active power response curve decays to steady state the fastest, proving the effectiveness of the proposed optimization method.

## Conclusion and perspective

In this paper, a virtual inertia optimization allocation method, which considers the small disturbance stability of the grid, is proposed. In addition, an optimal solution algorithm is developed considering the damping ratio sensitivity. The effectiveness of the proposed method is verified by time-domain simulations. This paper demonstrates that new techniques, such as virtual inertia control in systems with high proportional turbine penetration, can allow a system to adjust inertia and damping levels. The results indicate that the system damping ratio decreases with the inertia coefficient, thus affecting the system power angle stability. Sensitivity to parameters using damping ratios, justifying configurations with larger inertia and damping in wind farms with high capacity. Finally, the proposed selection method of optimal parameters for virtual inertia and damping can be used to maximize the small disturbance stability of a system under constraints. The arithmetic examples verify the effectiveness of the proposed selection method. The results show that the properly selected parameters can significantly improve the small disturbance stability while keeping the system's power angle stable.

Due to the limitation of the existing analysis methods, the effect of virtual inertia and its distribution characteristics on the small disturbance stability of a power system cannot be clearly explained. Therefore, this paper analyzes the influence of the virtual inertia distribution on the small disturbance stability of the system from an empirical point of view and provides a reference for the selection of capacity and parameters of the virtual inertia control. Based on the proposed optimization model and method, constraints and condition parameters can be extended. However, a quantitative description of the mechanism of the influence of damping and inertia control loops on the stability of small disturbances in DFIG containing power systems deserves further study.

## Supplementary Information

Supplementary Information.

## Data Availability

The datasets generated and/or analysed during the current study are available in the [NAME] repository, [PERSISTENT WEB LINK TO DATASETS]. Reprints and permissions information is available at www.nature.com/reprints. The authors confirm that the data supporting the findings of this study are available within the article and its supplementary materials.
