# Supplementary material for: Effect of DFIG control parameters on small signal stability in power systems
Source: Sci Rep. 2023 Feb 11;13:2476. doi: 10.1038/s41598-023-29278-5 (PMC9922310; doi:10.1038/s41598-023-29278-5)
Supplement: Supplementary file 1 — Supplementary Information. [file 41598_2023_29278_MOESM1_ESM.docx]

**APPENDIX A**

The stator of a doubly-fed wind turbine is directly connected to the network. The transient processes associated with the network are usually considered to decay very rapidly in electromechanical transient analysis, so ignoring the stator transients ensures consistency between models [2]. On this basis, a doubly-fed wind turbine model suitable for small disturbance analysis can be established, mainly considering the rotor current, rotor-side converter, and the transient characteristics of the mechanical parts of the doubly-fed wind turbine. Among them, the transient behavior of the rotor current of the doubly-fed wind turbine can be expressed as:

  $\frac{\text{d}I_{d\text{r}}}{\text{d}t}=-\frac{\omega_{\text{s}}}{T_{\text{r}}^{'}}I_{d\text{r}}-\frac{\omega_{\text{s}}}{X_{\text{r}}^{'}}\hat{U}_{d\text{r}}$ (A1)

$\frac{\text{d}I_{q\text{r}}}{\text{d}t}=-\frac{\omega_{\text{s}}}{T_{\text{r}}^{'}}I_{q\text{r}}-\frac{\omega_{\text{s}}}{X_{\text{r}}^{'}}\hat{U}_{q\text{r}}$ (A2)

$\hat{U}_{d\text{r}}=U_{d\text{r}}-sX_{\text{r}}^{'}I_{q\text{r}}$ (A3)

$\hat{U}_{q\text{r}}=U_{q\text{r}}+sX_{\text{r}}^{'}I_{d\text{r}}-s\frac{X_{\text{m}}}{X_{\text{s}}}\Psi_{\text{s}}$ (A4)

Where: Idr, Iqr are the rotor d, q-axis currents (constant q-axis direction coincides with the port voltage phase); ωs is the simultaneous rotational speed; T′r, X′r are the rotor time constant and reactance, respectively; Udr, Uqr are the intermediate variables introduced by the stator magnetic vector control for decoupling control; Udr, Uqr are the d, q-axis components of the rotor voltage, respectively; Ψs is the stator magnetic chain; Xm is the stator-rotor mutual inductance; Xs is the stator reactance; and s is the rotation difference rate.

The model of the rotor-side converter ignores the electromagnetic transient process of its inverter and focuses on its control link

$$\begin{matrix} \frac{\text{d}I_{d\text{ rref }}}{\text{d}t}=K_{1d}U_{\text{t}}\frac{X_{m}}{X_{\text{s}}}\left(\frac{\omega_{\text{s}}}{T_{\text{r}}^{'}}-\frac{1}{T_{1d}}\right)I_{d\text{r}}+ \\ K_{1d}U_{\text{t}}\frac{X_{\text{m}}}{X_{\text{s}}}\frac{\omega_{\text{s}}}{X_{\text{r}}^{'}}\widehat{U_{d\text{r}}}-\frac{K_{1d}}{T_{1d}}\left(\frac{U_{\text{t}}^{2}}{X_{\text{s}}}+Q_{\text{sref}}\right)\text{············································}(\text{A}5) \end{matrix}$$

$\frac{\text{d}\hat{U}_{d\text{r}}}{\text{d}t}=\frac{K_{2d}}{T_{2d}}\left(I_{d\text{r}}-I_{d\text{rref}}\right)-K_{2d}\left(\frac{\omega_{\text{s}}}{T_{\text{r}}^{'}}I_{d\text{r}}+\frac{\omega_{\text{s}}}{X_{\text{r}}^{'}}\hat{U}_{d\text{r}}\right)$ (A6)

$$\begin{matrix} \text{ }\frac{\text{d}I_{q\text{rref}}}{\text{d}t}=K_{1q}U_{\text{t}}\frac{X_{\text{m}}}{X_{\text{s}}}\left(\frac{\omega_{\text{s}}}{T_{\text{r}}^{'}}-\frac{1}{T_{1q}}\right)I_{q\text{r}}+ \\ \text{ }K_{1q}U_{\text{t}}\frac{X_{\text{m}}}{X_{\text{s}}}\frac{\omega_{\text{s}}}{X_{\text{r}}^{'}}\hat{U}_{q\text{r}}-\frac{K_{1q}}{T_{1q}}P_{sref}\text{······································}(\text{A}7) \end{matrix}$$

  $\frac{\text{d}U_{q\text{r}}}{\text{d}t}=\frac{K_{2q}}{T_{2q}}\left(I_{q\text{r}}-I_{q\text{rref}}\right)-K_{2q}\left(\frac{\omega_{\text{s}}}{T_{\text{r}}^{'}}I_{q\text{r}}+\frac{\omega_{\text{s}}}{X_{\text{r}}^{'}}\hat{U}_{q\text{r}}\right)$ (A8)

Where: Idrref, Iqrref are the rotor d, q axis current reference values respectively; Ut is the stator voltage; Psref, Qsref are the active and reactive power setting values; K1d, T1d, K1q, T1q, K2d, T2d, K2q, T2q are the controller parameters.

The mechanical characteristics of doubly-fed wind turbines are an essential factor affecting the stability of the system with small disturbances. This paper uses a two-mass block model to characterize the mechanical part of a doubly-fed wind turbine. The mechanical part of a doubly-fed wind turbine.

  $2H_{\text{gen}}\frac{\text{d}s}{ \text{d}t}=-\frac{X_{\text{m}}}{X_{\text{s}}}U_{\text{t}}I_{\text{qr}}-T_{\text{shaft }}$ (A9)

$2H_{\text{t}}\frac{\text{d}\omega_{\text{tur }}}{\text{d}t}=T_{\text{tur }}-T_{\text{shaft }}$ (A10)

$\frac{\text{d}\theta_{\text{shaft }}}{\text{d}t}=\omega_{\text{tur }}-\omega_{0}(1-s)$ (A11)

$T_{\text{shaft }}=K_{\text{shaft }}\theta_{\text{shaft }}+D_{\text{shaft }}\left[\omega_{\text{tur }}-\omega_{0}(1-s)\right]$ (A12)

where: Hgen and Ht are the rotor and wind turbine inertia constants, respectively. Tshaft is the torque provided by the gearbox; Ttur is the torque provided by the wind turbine; ωtur is the wind turbine speed; ω0 is the synchronous speed; θshaft is the drive shaft torsion angle; Kshaft is the drive shaft stiffness; and Dshaft is the torsion damping coefficient.

**APPENDIX B**

The impedance of the transformer connected to the synchronous generator is 0+j0.015pu at 900MVA and 20/230kV with a ratio of 1.0; the impedance of the transformer connected to the wind farm is 0+j0.015pu at 900MVA and 0.69/230kV with a ratio of 1.0. Using Gen2 as the reference motor, the rest of the synchronous generation active output is 612MW. The synchronous generator parameters are shown in Table B1, and the excitation system parameters are shown in Table B3. The single double-fed turbine parameters are shown in Table B4

The reactive power supplied by the load and the shunt capacitors at nodes 7 and 9 are as follows.

$$\begin{matrix} P_{L7}=567MW, Q_{L7}=100Mvar,Q_{C7}=200Mvar \\ P_{L9}=2190MW,Q_{L9}=100Mvar,Q_{C9}=300Mvar \end{matrix}$$

Tab. B1 Parameters of synchronous generator

| Parameters | | Value | Parameters | Value | Parameters | Value |
| --- | --- | --- | --- | --- | --- | --- |
| *S*/MVA | | 900 | *X*_d_"/pu | 0.25 | *T*_q0_"/s | 0.05 |
| *U*/kV | | 20 | *X*_q_"/pu | 0.25 | *H*_Gen1_/s | 6.5 |
| *X*_d_/pu | | 1.8 | *X*_1_/pu | 0.2 | *H*_Gen2_/s | 6.5 |
| *X*_q_/pu | | 1.7 | *T*_d0_'/s | 8.0 | *H*_Gen3_/s | 6 |
| *X*_d_'/pu | | 0.3 | *T*_q0_'/s | 0.4 | *H*_Gen4_/s | 6 |
| *X*_q_'/pu | 0.55 | | *T*_d0_"/s | 0.03 | *D* | 0 |

Tab. B2 Parameters and controllers for DFIG

| Parameters | Value | Parameters | Value |
| --- | --- | --- | --- |
| *S*/MVA | 2.0 | *K*_i1_ | 40 |
| *P*/MW | 3.6 | *K*_p2_ | 0.0496 |
| *r*_s_/pu | 0.004499 | *K*_i2_ | 3.875 |
| *L*_s_/pu | 0.09241 | *K*_p3_ | 4.0 |
| *r*_r_/pu | 0.0549 | *K*_i3_ | 40 |
| *L*_r_/pu | 0.09955 | *K*_p4_ | 0.0496 |
| *L*_m_/pu | 3.95279 | *K*_i4_ | 3.875 |
| *K*_p1_ | 4.0 | *T*_w_ | 0.01 |
